# Quantification of Radiation-Induced Fibrosis in Head and Neck Cancer Patients Using Shear Wave Elastography

**DOI:** 10.7759/cureus.71159

**Published:** 2024-10-09

**Authors:** Bryan Renslo, Rahul Alapati, Joseph Penn, Katherine M Yu, Shiloh Sutton, Celina G Virgen, Tuleen Sawaf, Kevin J Sykes, Sufi M Thomas, Frank T Materia, Jill A Jones, Andres Bur

**Affiliations:** 1 Otolaryngology - Head and Neck Surgery, University of Kansas Medical Center, Kansas City, USA; 2 Diagnostic Radiology, University of Kansas Medical Center, Kansas City, USA; 3 Otolaryngology - Head and Neck Surgery, University of Kansas School of Medicine, Kansas City, USA

**Keywords:** head and neck, radiation fibrosis, radiation therapy, shear wave elastography, squamous cell carcinoma

## Abstract

Background

Radiation-induced fibrosis (RIF) is a common side effect in head and neck cancer (HNC) patients treated with radiotherapy. A validated tool to measure RIF does not currently exist. In this study, we evaluate the ability of shear wave elastography (SWE) to measure RIF in HNC survivors.

Methods

HNC patients treated with surgery and adjuvant radiation between January and September 2022 at a single tertiary medical center were enrolled with age and gender-matched control patients. Median tissue stiffness was recorded with SWE at the sternocleidomastoid (SCM) muscle, overlying subcutaneous tissues (ST), and the base of the tongue (BOT).

Results

A total of 20 patients were included. Tissue stiffness was significantly higher among HNC patients at the SCM ipsilateral (53.7 kPa vs. 17.5 kPa, p=0.002) and contralateral (44.1 kPa vs. 21.6 kPa, p=0.027) than the side of treatment. Tissue stiffness was not significantly different in ST ipsilateral (6.6 kPa vs. 5.3 kPa, p=0.922) or contralateral (8.0 kPa vs. 6.4 kPa, p=0.426) to treatment. Tissue stiffness at the BOT was not significantly different (19.1 kPa vs. 13.1 kPa, p=0.084).

Conclusion

SWE is a noninvasive method for measuring tissue fibrosis and is a promising tool for the measurement of RIF in patients with HNC. SWE measurements were taken from the SCM, ST, and BOT in HNC patients at least one year out from treatment with surgery and adjuvant radiation as well as age and gender-matched control patients. HNC patients showed significantly higher levels of tissue stiffness at the SCM both ipsilateral and contralateral to radiotherapy. SWE may be useful for tracking the progression of RIF in HNC patients.

## Introduction

Selected high-risk patients with head and neck squamous cell carcinoma are treated with adjuvant radiation therapy following surgery in an effort to improve locoregional control and survival [1]. Repetitive injury from radiation at doses greater than 60 Gy leads to radiation-induced fibrosis (RIF), which is the development of scar tissue in skin and musculature in response to chronic inflammation induced by cellular death [2]. RIF can cause debilitating symptoms, including dysphagia and pain, that can have a significant impact on patients’ quality of life [3,4].

Currently, there is no standard measurement of RIF in patients after treatment for head and neck cancer (HNC) [5]. Clinically, RIF is subjectively evaluated based on symptomatology and physical exam [6]. Histologic quantification of fibrosis, as utilized for liver and lung fibrosis, would be a more objective estimate of disease; however, this would require an invasive biopsy for diagnosis [7,8]. Patient questionnaires that attempt to quantify patient symptoms of dysphagia, such as the MD Anderson Dysphagia Inventory (MDADI) or the European Organization for Research and Treatment of Cancer (EORTC) Quality of Life Questionnaire, are not specific to RIF [9]. Given that RIF tends to progress well after the completion of therapy, there is a need for more objective and minimally invasive quantification methods to allow for early treatment intervention.

Shear wave elastography (SWE) offers a noninvasive method for quantitatively estimating fibrosis by measuring the elasticity of tissues subjected to an acoustic force. SWE has been widely researched across a range of organ systems, including the liver, spleen, and a variety of soft tissues [10-13]. However, studies specific to RIF in HNC patients from the international literature are sparse [14,15]. The aim of the present study is to determine the feasibility of using SWE to quantify RIF in HNC patients treated with surgery and adjuvant radiotherapy. In doing so, SWE may serve as a measurement tool to quantify RIF, monitor its progression, and evaluate the effectiveness of therapies aimed at reducing RIF after treatment.

## Materials and methods

Study design and population

This prospective feasibility study was performed at a single tertiary institution (University of Kansas Medical Center, Kansas City, KS). Patients who underwent primary surgery and adjuvant radiotherapy for HNC and were at least one year out from the end of treatment were identified and enrolled between January 2022 and September 2022. Patients were excluded if they had neoadjuvant treatment or a preceding history of radiotherapy. Gender and age-matched control patients (within 10 years) were prospectively identified and enrolled during the same time period. Control patients had no history of cancer, head and neck radiotherapy, or head and neck pathology. Background demographic and clinical data were collected for each patient. Patients were also administered the MDADI [16] and the EORTC [17] at the time of consent. The study was approved by the Institutional Review Board of the University of Kansas Medical Center.

Ultrasound examination

Ultrasound imaging was performed by a single sonographer (SS) with six years of SWE experience. All exams were performed on a commercially available GE LOGIQ E10 unit (GE HealthCare, Waukesha, WI) with a linear array 6-15 MHz transducer. Patients were imaged in the supine position on a flat examination bed. A thin pillow was placed behind the shoulders to allow relaxed neck extension, and the neck was straight at midline. A gel standoff was utilized to minimize transducer pressure.

**Figure 1:**
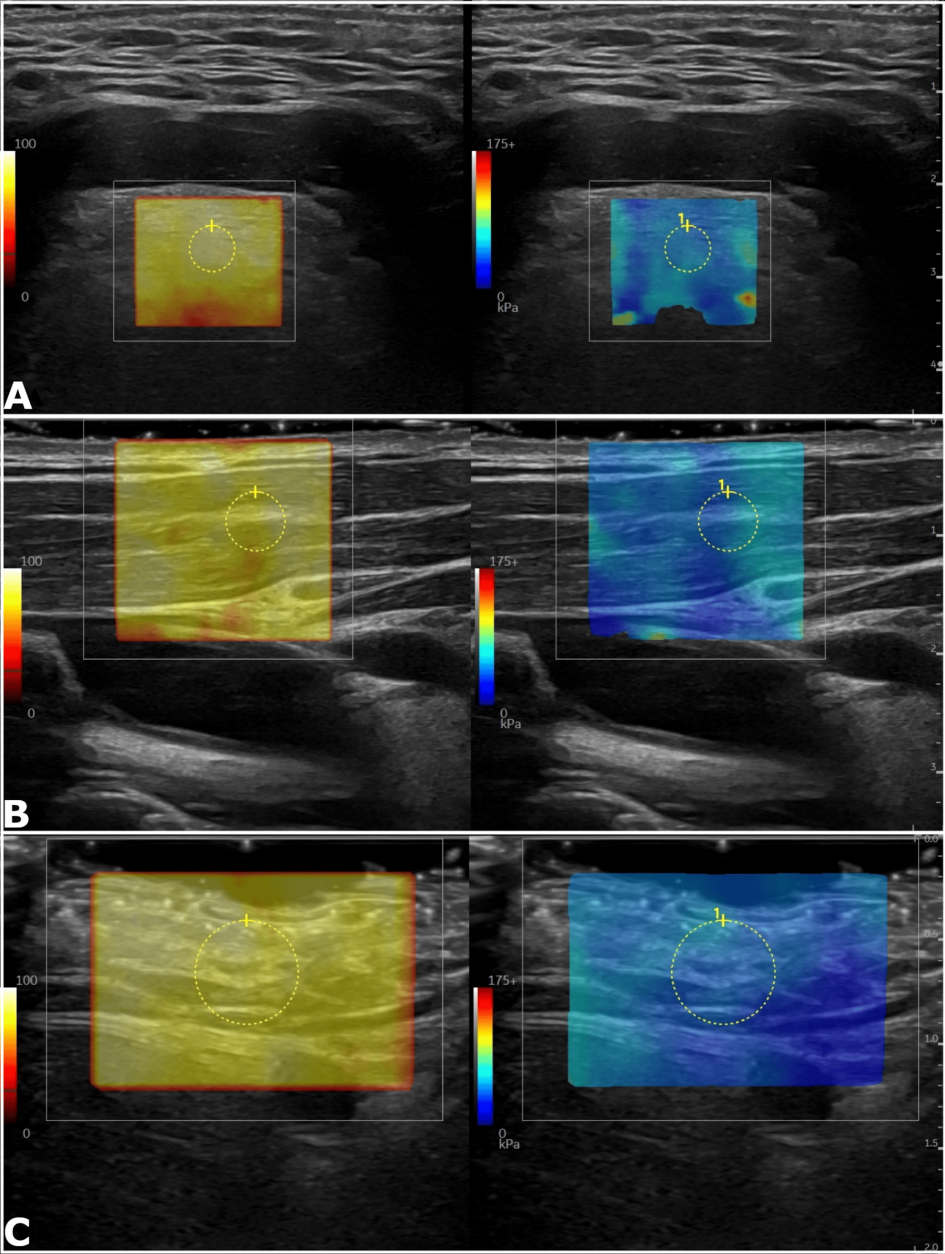
Parts A-C. Representative 2D shear wave elastography in control tissue. Sample images were obtained at the base of the tongue (A), right sternocleidomastoid (B), and right soft tissues (C). Sample elasticity measurements were 13.51 kilopascals (kPa), 13.27 kPa, and 10.65 kPa, respectively. The left image is the corresponding quality indicator map and the right image displays the relative tissue elasticity.

**Figure 2:**
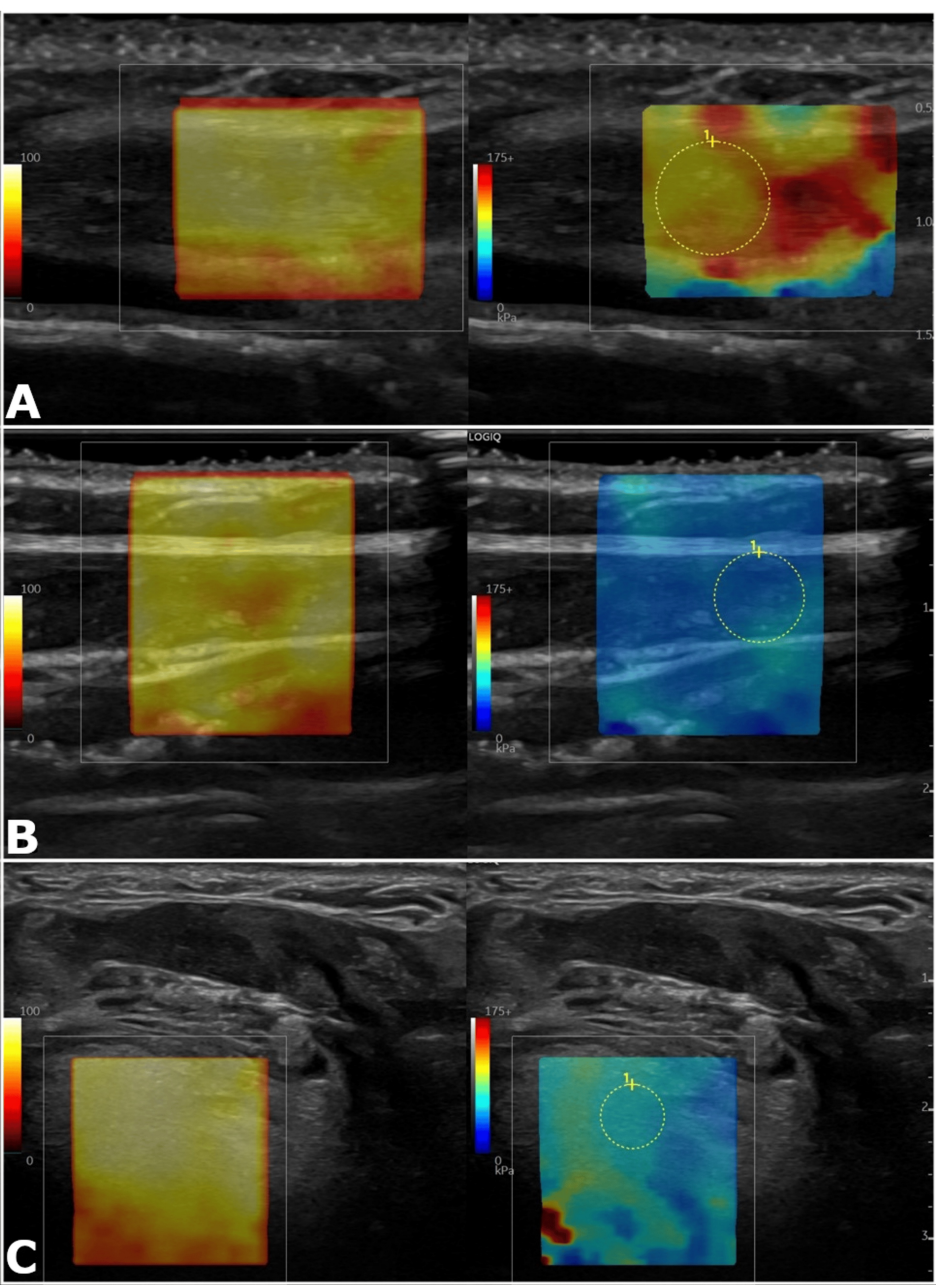
Parts A-C. Representative 2D shear wave elastography in a patient with head and neck cancer treated with adjuvant radiotherapy. Sample images obtained at the left sternocleidomastoid on the side of treatment (A), right sternocleidomastoid contralateral to treatment (B), and base of the tongue (C). Sample elasticity measurements were 81.54 kilopascals (kPa), 12.81 kPa, and 24.17 kPa, respectively. The left image is the corresponding quality indicator map and the right image displays the relative tissue elasticity.

Measurements were obtained in five anatomic locations: bilateral mid sternocleidomastoid muscle (SCM), bilateral subcutaneous tissues (ST) immediately adjacent to the SCM, and the base of the tongue (BOT). For measurements of the SCM, the muscle was positioned at 90 degrees to minimize anisotropy, and the transducer was oriented along the longitudinal axis of the mid-muscle belly perpendicular to the surface of the skin. The ST measurements were obtained in the superficial tissues overlying or immediately adjacent to the SCM. This included subcutaneous tissue and fat superficial to the platysmal layer. For measurements of the BOT, the transducer was placed at the midline in the submental region and directed cephalad to the central genioglossus muscle. The measurement region of interest (ROI) was consistent at 5 mm diameter on all images. A representative image from each measurement location is shown (Figures 1-2). Ten measurements were obtained in each location, and median stiffness values were reported in kilopascals (kPa). The reliability of measurements was assessed using the IQR/median ratio, which has previously been utilized as criteria for SWE measurement reliability (very reliable: IQR/median<0.10, reliable: 0.10<IQR/median<0.30; poorly reliable: IQR/median>0.30) [18-20].

Statistical analysis

Subject data were paired with matched controls for statistical analysis. Values ipsilateral and contralateral to the side of radiation were analyzed separately. In patients who had bilateral radiation therapy, both sides were considered ipsilateral. Variables were compared using a two-sided paired Wilcoxon signed-rank test for continuous data and Pearson’s chi-squared test for categorical variables. Correlation analysis was conducted utilizing Pearson’s correlation test. An α threshold of α = 0.05 was used for all unique comparisons. All statistical analysis was conducted in R (version 4.2.1; R Development Core Team, Vienna, Austria).

## Results

A total of 20 subjects were included for analysis, with 10 HNC patients and 10 age and gender-matched controls. The average age of all participants was 65.7±11.4 years, and six (30%) patients were female. There were no significant differences in demographics between the two cohorts (Table 1). For the purposes of this study, sex refers to biological designation at birth. HNC patients scored significantly lower on the MDADI (3.2 vs. 4.9, p=0.004) and significantly higher on the EORTC (1.8 vs. 1.2, p=0.006) (Table 1).

**Table 1:** Paired testing between head and neck cancer and control patient cohorts. HNC, head and neck cancer; CI, Confidence interval; MDADI, MD Anderson Dysphagia Inventory; EORTC, European Organization for Research and Treatment of Cancer Quality of Life Questionnaire Head and Neck Module; kPa, kilopascals; SCM, sternocleidomastoid; IQR, interquartile range; med, median; ST, soft tissue. Statistics calculated using the paired Wilcoxon signed rank-sum test for continuous data and Pearson's chi-squared test for categorical data. ^1^Mean (SD), n (%); ^2^Paired Wilcoxon signed rank-sum test, Pearson's chi-squared test; ^3^W, χ2

Characteristic	Overall, N = 20^1^	Control, N = 10^1^	HNC, N = 10^1^	p-value^2^	Test Statistic^3^
Age	65.7 (11.4)	64.1 (10.2)	67.3 (12.9)	0.058	6.0
Sex, female	6 (30.0%)	3 (30.0%)	3 (30.0%)	>0.999	0.0
Smoking Status				0.139	3.94
Current	5 (25.0%)	1 (10.0%)	4 (40.0%)		
Former	7 (35.0%)	3 (30.0%)	4 (40.0%)		
Never	8 (40.0%)	6 (60.0%)	2 (20.0%)		
Survey Results					
MDADI	4.0 (1.1)	4.9 (0.2)	3.2 (0.9)	0.004	54.0
EORTC	1.5 (0.5)	1.2 (0.2)	1.8 (0.4)	0.006	0.0
Elastography Results (kPa)					
Ipsilateral SCM	35.6 (26.2)	17.5 (9.3)	53.7 (25.2)	0.002	0.0
Ipsilateral SCM (IQR/med)	0.290 (0.134)	0.260 (0.153)	0.321 (0.111)	0.275	16.0
Contralateral SCM	32.9 (20.0)	21.6 (14.8)	44.1 (18.6)	0.027	4.0
Contralateral SCM (IQR/med)	0.242 (0.131)	0.199 (0.095)	0.285 (0.153)	0.074	7.0
Ipsilateral ST	6.0 (4.0)	5.3 (2.0)	6.6 (5.4)	0.922	29.0
Ipsilateral ST (IQR/med)	0.401 (0.257)	0.304 (0.187)	0.499 (0.288)	0.232	15.0
Contralateral ST	7.2 (3.1)	6.4 (1.9)	8.0 (4.0)	0.426	15.0
Contralateral ST (IQR/med)	0.274 (0.169)	0.271 (0.188)	0.278 (0.159)	0.426	15.0
Base of Tongue	16.1 (6.8)	13.1 (3.6)	19.1 (8.0)	0.084	10.0
Base of Tongue (IQR/med)	0.287 (0.196)	0.305 (0.238)	0.270 (0.154)	>0.999	27.0

On SWE, HNC patients showed significantly higher levels of stiffness at the SCM in both ipsilateral (53.7 kilopascals vs. 17.5 kPa, p=0.002) and contralateral (44.1 kPa vs. 21.6 kPa, p=0.027) to the side of radiotherapy (Table 1). Tissue stiffness at the BOT was not significantly higher in HNC patients (19.1 kPa vs. 13.1 kPa, p=0.084) (Table 1). Tissue stiffness was not significantly higher among HNC patients at the ipsilateral (6.6 kPa vs. 5.3 kPa, p=0.922) or contralateral (8.0 kPa vs. 6.4 kPa, p=0.426) ST (Table 1). Of note, the reliability of measurements based on IQR/median were on average reliable at the ipsilateral SCM, contralateral SCM, contralateral ST, and BOT (Table 1).

**Figure 3:**
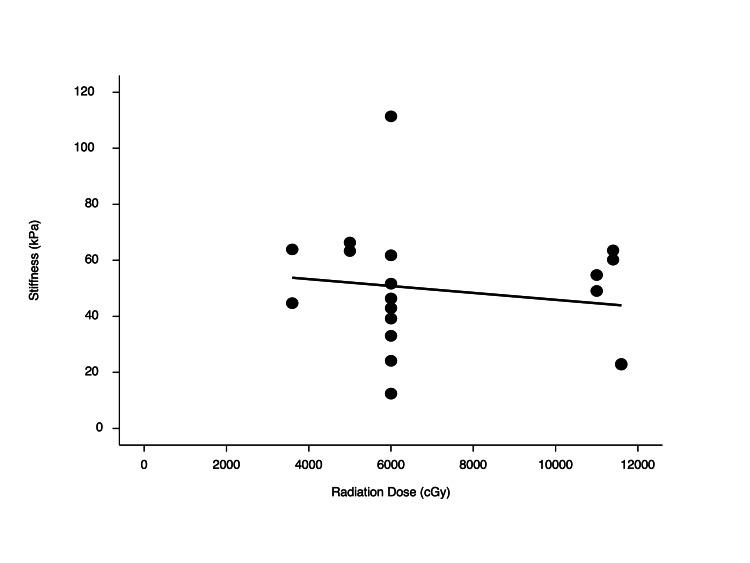
Radiotherapy dose versus tissue stiffness. Tissue stiffness per shear wave elastography was not significantly correlated with the dose of radiation (R=-0.16, p=0.512). kPa, kilopascal; cGy, centigray

Among HNC patients, nine (90%) had oral cavity cancer, and one (10%) had oropharyngeal cancer (Table 2). Of the HNC patients, nine were treated with neck dissection at the time of surgery, none of which included modified radical neck dissection with removal of the SCM. Half of the patients had nodal metastatic disease, and none of the patients had distant metastases. The median time from the end of treatment to SWE measurement was 48 months (IQR = 42.75). Additional clinical information for the HNC cohort can be found in the appendix (Table 3). No correlation was found between radiotherapy dose and tissue stiffness on SWE (R=-0.16, p=0.512) (Figure 3).

**Table 2:** Head and neck cancer patient clinical summary. ^1^n (%)

Characteristic	N = 10^1^
Site	
Oral Cavity	9 (90.0%)
Oropharynx	1 (10.0%)
T stage	
T1	0 (0.0%)
T2	5 (50.0%)
T3	3 (30.0%)
T4	2 (20.0%)
N stage	
N0	5 (50.0%)
N1	1 (10.0%)
N2	4 (40.0%)
N3	0 (0.0%)
M stage	
MX	1 (10.0%)
M0	9 (90.0%)
M1	0 (0.0%)
Overall stage	
I	1 (10.0%)
II	2 (20.0%)
III	2 (20.0%)
IV	5 (50.0%)

## Discussion

Intensity-modulated radiotherapy (IMRT) has become the standard of care for HNC radiotherapy in recent years [21]. Despite IMRT having a better side effect profile than conventional radiotherapy, it may still leave patients with debilitating acute and late toxicities that have been attributed to RIF [3,22]. Acute toxicities include insomnia, appetite loss, coughing, mucositis, dysgeusia, pain, and dysphagia. Late toxicities, defined as greater than 90 days after treatment cessation, include similar symptoms with the additional concern for xerostomia, trismus, and atrophy [23].

RIF is observed in over 50% of HNC patients treated with radiotherapy from one to eight years post treatment and is associated with high morbidity [4]. For this reason, having objective measurements of fibrosis in at-risk patients would be valuable to oncologists. By monitoring fibrosis development, patients can be selected to receive treatment aimed at limiting radiation toxicity. Current treatment strategies for head and neck fibrosis include mechanical, topical, and systemic treatment options [24]. Mechanical maneuvers are a preventative option against the development of trismus and also help treat lymphedema [25-27]. Pentoxifylline with or without vitamin E is an immunomodulatory drug that has shown promise in reducing fibrosis levels through the improvement of microvasculature, downregulation of cytokines, and inhibition of TGF-β [28-31]. Statins also inhibit the fibrotic signaling mechanism, and a daily dose of pravastatin 40 mg for one year was shown to decrease skin thickness in HNC patients with cutaneous and subcutaneous fibrosis [32]. Estimation of tissue stiffness by SWE could also serve as a quantitative tool to measure the effectiveness of treatment regimens.

Prior studies in other tissue types have quantified fibrosis through histologic means. In liver fibrosis, multiple grading systems have been proposed based on the location and severity of collagen deposition relative to the portal system [7]. Similar grading systems have been proposed for lung fibrosis [8]. These grading systems are only semi-quantitative and require invasive biopsies. SWE is advantageous as it provides a non-invasive method to diagnose and monitor pathologies that cause increased tissue stiffness throughout the body. Elasticity is the tendency of tissue to resist deformation with an applied force, or the resumption of its original shape after the force has been removed. With increased elasticity, the speed at which waves can propagate through the tissue decreases. As such, by applying dynamic stress such as an acoustic radiation force to tissue and measuring the speed of wave propagation, the stiffness of the tissue can be quantified [10,33]. Increased tissue stiffness may be a result of tissue inflammation, edema, fibrosis, or a combination of these etiologies. Therefore, SWE results should be cautiously interpreted with regard to all patient factors. We enrolled patients in the HNC arm who were at least one year from the end of adjuvant radiotherapy to allow for the resolution of acute radiation toxicities, such as dermatitis and mucositis. Similarly, the estimation of RIF may be confounded in patients who have had recent surgeries, infection, or metastatic disease.

A study previously conducted by Liu et al. found that SWE measurements at the SCM and overlying ST were significantly higher in patients treated with head and neck radiation as compared to healthy control patients [15]. The present study similarly found increased stiffness at the SCM, but non-significant differences in stiffness in the ST. We found that measurements obtained in the ST of all patients tended to be highly variable and showed poor-quality maps. This is likely due to the relatively small target area and superficial location, which may lead to inhomogeneity of the elastography image [10,11]. In comparison, the SCM was easily identifiable and of sufficient size to facilitate the placement of the SWE measurement ROI in all patients. The SCM would therefore be a good target for monitoring RIF, even in patients who may have muscular atrophy or with novice sonographers. These findings are reflected in the IQR/median ratios, where SCM measurements were more reliable than ST measurements on average. It should be noted, however, that SWE reliability criteria were established in the measurement of liver stiffness [18-20]. Given differences in tissue and pathology, these criteria may not be as appropriate for head and neck measurements.

Our study further compared tissue stiffness values both ipsilateral and contralateral to the site of radiation. Patients treated with radiotherapy showed a statistically significant increase in SCM stiffness on both sides when compared to control patients. Radiation target volumes are intentionally created slightly larger than the tumor size to account for microscopic tumor extension and random set-up errors during treatment [34]. Our findings are likely to be the result of radiation crossover from larger dose fields, resulting in some degree of fibrosis on the contralateral side. There is also potential for radiation to lead to changes in muscle mechanics and compensatory hypertrophy of contralateral normal tissues. While this study did find increased SWE measurements in radiotherapy-treated patients overall, a dose-response relationship was not established. A repeat study with a larger cohort of patients would help determine if such a relationship exists.

This study also evaluated the stiffness of the base of tongue, which trended higher for HNC patients, but was not statistically significant. The majority of our patient cohort had primary oral cavity cancer as opposed to other tumor sites, which may have affected our results. Specifically, the base of tongue is typically outside of the high-dose radiation field for patients with oral cavity cancer. However, imaging of the tongue also comes with some inherent technical challenges as the tongue is not externally visible during imaging. It would be difficult to control for variations in tongue positioning between patients. Further studies with larger sample sizes and patients separated into cohorts based on tumor site would help clarify these findings.

There are several features specific to skeletal muscles that will impact the accuracy and reliability of SWE measurements. In particular, muscle contraction will result in increased stiffness values [35]. Measurements should be obtained with the patient in a relaxed state and neutral position to minimize variation between subjects. Anisotropy within skeletal muscles has been shown to result in variable SWE measurements based on the orientation of muscle fibers relative to the angle of the ultrasound probe [34-36]. In this study, the sonographer aligned the SCM 90 degrees relative to the angle of insonation to minimize anisotropy artifact. However, the genioglossus muscle is unique in that the muscle fibers are oriented in a fan-like array, making it highly prone to anisotropic variations. Pre- and post-processing techniques have been investigated to compensate for anisotropic effects, although this continues to be a limiting factor in the application of skeletal muscle SWE [35,36]. Patient factors such as age, sex, and BMI, as well as environmental factors, such as room or muscle temperature, can also confound results [32]. In our study, we attempted to minimize these effects by utilizing age and gender-matched patients who would have similar body habitus. Future studies should take other factors into account, including BMI and exam setting.

Limitations

While this study demonstrates the potential application of SWE to quantitatively measure RIF in patients treated for head and neck cancer, it has several limitations. This was a small, single-center cohort study primarily limited by sample size. SWE is highly operator-dependent, and good quality control is needed to ensure that SWE measurements are valid; therefore, the results may not be generalizable to all practices. Variability in technique, tools (including a gel standoff), and pressure applied during measurement may affect results. To mitigate these variables, a single sonographer obtained all measurements using a consistent technique. The correlation between SWE measurements and radiation dose, dysphagia, and pain would further establish the utility of SWE; however, these comparisons were not possible given our small sample size. Furthermore, SWE measurements were taken from HNC patients at least one year post treatment with adjuvant radiotherapy. Longitudinal measurements at baseline and throughout radiation treatment may reveal progressive changes in scar tissue development that were not measured by this feasibility study. Additionally, further studies should include patients treated with definitive chemoradiotherapy. The HNC cohort is limited somewhat by selection bias in that all patients were identified at least 16 months out from treatment as doing fairly well clinically. This study was not designed to study the clinical relevance of RIF but more to provide proof of concept regarding the utility of SWE, so this should not affect the primary objective of the study.

## Conclusions

RIF is a common side effect in HNC patients treated with radiotherapy and is associated with debilitating symptoms, including dysphagia and pain. SWE is a noninvasive method for measuring tissue fibrosis and is a promising tool for the measurement of RIF in patients with HNC. SWE measurements were taken from the SCM, ST, and BOT in HNC patients at least one year out from treatment with surgery and adjuvant radiation, as well as age and gender-matched control patients. HNC patients showed significantly higher levels of tissue stiffness at the SCM both ipsilateral and contralateral to radiotherapy. Tissue stiffness of the ST and BOT were not significantly different between the two groups. The application of SWE for HNC patients may prove useful in identifying those most at risk for adverse symptoms and those who may benefit from fibrosis therapy options. Further study with additional longitudinal measurements throughout treatment and with a larger sample size is warranted.

## Appendices

**Table 3:** Head and neck cancer patient data HPV, human papillomavirus; cGy, centigrey; SWE, shear wave elastography; F, female; M, male

							Stage			Radiation Data		
Patient	Age	Sex	Smoking	Pack Years	HPV	Tumor Site	T	N	M	Dose (cGy)	Fractions	Time to SWE (months)
1	71	F	Current	106	Negative	Oral Cavity	3	0	0	5000	25	42
2	66	M	Current	75	Positive	Oral Cavity	4	2	0	6000	30	19
3	82	M	Former	10	Unknown	Oral Cavity	4	2	0	6000	30	54
4	73	F	Never		Positive	Oropharynx	2	2	0	11400	60	87
5	34	M	Former	5	Negative	Oral Cavity	2	0	0	11600	30	16
6	67	M	Former	10	Negative	Oral Cavity	3	1	0	6000	30	65
7	75	M	Never		Negative	Oral Cavity	2	2	0	11000	55	59
8	63	M	Current	50	Negative	Oral Cavity	2	0	x	3600	18	60
9	74	M	Current	45	Unknown	Oral Cavity	3	0	0	6000	30	17
10	68	F	Former	44	Unknown	Oral Cavity	2	0	0	6000	30	28
